# Auxins in the right space and time regulate pea fruit development

**DOI:** 10.1093/jxb/erac237

**Published:** 2022-06-24

**Authors:** Jutta Ludwig-Müller

**Affiliations:** Technische Universität Dresden, Faculty of Biology, D-01062 Dresden, Germany

**Keywords:** Auxin biosynthesis, auxin receptor, 4-chloro-indole-3-acetic acid, ethylene, gibberellin, pea reproductive development, *Pisum sativum*

## Abstract

This article comments on:

**Ozga JA, Jayasinghege CPA, Kaur H, Gao L, Nadeau CD, Reinecke DM**. 2022 Auxin receptors as integrators of developmental and hormonal signals during reproductive development in pea. Journal of Experimental Botany **73**, 4094–4112

**The growth hormone auxin has been implicated as a necessary factor in the development of different types of fruit. The perception of auxin is essential, especially in pea plants where two types of auxin are present, and the chlorinated form seems to be the more active one. The elegant and detailed work by Ozga *et al.* (2022** **) has shown how indole-3-acetic acid (IAA), 4-chloro-indole-3-acetic acid (4-Cl-IAA), gibberellin (GA), and ethylene contribute to pea seed development by dissecting the different tissues for the analysis of the response in addition to the interaction of pea auxin receptors with the Aux/IAA repressors, and identified a complex tissue-specific hormonal network.**

A tissue-specific complex hormonal network regulates pea pod (pericarp) growth and seedling development. Previous work on pea seed development focused on both individual hormonal pathways, namely GAs (Ozga *et al.*, 2009; Nadeau *et al.*, 2011) or auxins alone (Reinecke *et al.*, 1999), and interactions between hormonal pathways (Ozga and Reinecke, 1999; Ozga *et al.*, 2003; Johnstone *et al.*, 2005). The interaction of growth hormones, auxins, and GAs, together with a negative regulator ethylene decide how and when pea pods develop. The role of the two major auxins, IAA and its chlorinated form 4-Cl-IAA, in pea development is still not well understood. The biosynthetic pathway for IAA in pea has been confirmed to involve the indole-3-pyruvic acid (IPyA) pathway (Tivendale *et al.*, 2012), which is comprised of the TAR and YUC enzymes known to be also active in other plant species (Mano and Nemoto, 2012) (Box 1). However, the step at which the chlorine is added still remains an open question. Since the enzymes are able to use tryptophan as well as 4-Cl-tryptophan, this has been taken as evidence that tryptophan needs to be chlorinated (Tivendale *et al.*, 2012). The analogy to bacterial halogenases suggested that tryptophan is the most likely target for halogenation (Patallo *et al.*, 2017), but up to now a halogenase for the 4-position of the indole moiety has not been identified.

Box 1. **Auxin biosynthesis and perception in pea**

Auxin biosynthesis and signalling in pea fruit and involvement in development of pods and seeds. While in the majority of plant species IAA is the most active auxin, in pea and other legumes it might be 4-Cl-IAA. This is indicated by the size of the structures. The biosynthesis in pea plants is mediated by two different enzymes, TAR1 and YUC1, in analogy to other plants. Auxin perception needs the interaction of the hormone with an F-box protein, TIR1, and the AFB family. The F-box protein is part of a ubiquitin ligase complex that ubiquitinates targets for degradation in the 26S proteasome. The target in the case of auxin signalling is a repressor of transcription (Aux/IAA proteins). If these bind to the transcriptional activators (ARF proteins), then the transcription is inhibited (left). Only upon higher auxin concentrations are the inhibitors recruited together with auxin to the F-box binding site and then ubiquitinated for subsequent degradation. The transcriptional activation via ARF proteins can then take place (right). Experimental evidence was reported for the physical binding of 4-Cl-IAA and IAA to all pea auxin receptors by Ozga *et al.* (2022). The pea plant cartoon is from BioRender (free version: https://biorender.com/; accessed 5.5.2022). The structures were taken from Wikipedia (https://en.wikipedia.org/wiki/4-Chloroindole-3-acetic_acid; https://en.wikipedia.org/wiki/Indole-3-acetic_acid; accessed 5 May 2022).

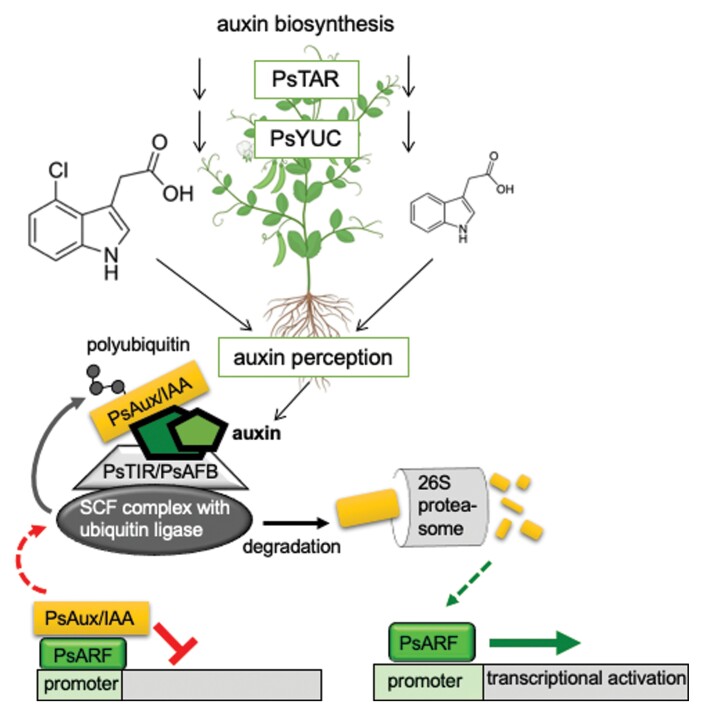

The set of auxin receptors that belong to the F-box protein class TIR1/AFB have been extensively characterized in *Arabidopsis thaliana* (Dharmasiri *et al.*, 2005; Kepinski and Leyser, 2005; Parry *et al.*, 2009). They need to interact with auxin—both natural and synthetic auxins are recognized by different family members—and a transcriptional inhibitor of the large Aux/IAA family (Overvoorde *et al.*, 2005). Only under these conditions is the Aux/IAA repressor polyubiquitinated by the ubiquitin ligase part of the protein complex (Box 1) and then degraded in the 26S proteasome of the cell. Transcription can start only then since the ARF-type activators can bind to the promoter of auxin-responsive genes (Ulmasov *et al.*, 1997). The analysis of the sequences for auxin perception in pea has revealed the occurrence of six genes that encode putative auxin receptors and potentially bind with higher affinity to 4-Cl-IAA than to IAA (Ozga *et al.*, 2022). The work by Ozga *et al.* (2022), being the latest research in a series of publications on the same topic (Johnstone *et al.*, 2005; Jayasinghege *et al.,* 2017, 2019), has tremendously advanced the field by combining different tissues and their dissection into smaller functional parts with different methods such as treatment with hormones, molecular interaction assays, and transcript and hormone analyses. Overall, the results of this challenging work show a developmental regulation of auxin receptor genes as well as genes for auxin biosynthesis and hormone levels in seedling and fruit tissues.

## Why is 4-Cl-IAA more active in pea tissues?

Why 4-Cl-IAA is more bioactive than IAA is a question that has formed the basis of a number of studies. In pea, the higher bioactivity of 4-Cl-IAA compared with IAA was recognized and studied already early on (Reinecke *et al.*, 1995; Magnus *et al.*, 1997); however, without having the possibility to knock out only one pathway by identifying the halogenation step, this still remained a point of discussion. Reinecke *et al.* (1999) first hypothesized that the binding to the receptor complex should favour the 4-Cl derivative using structure–activity studies, and this hypothesis is further supported by the receptor characterization in Ozga *et al.* (2022). Deseeded pericarps have been used to partially answer the question about the effect of the two auxins since they can be supplemented with either auxin and then the growth response analysed. It seems that 4-Cl-IAA is more stable, but it could also be binding with higher affinity than IAA to the receptors of those plants, where 4-Cl-IAA (but not IAA) plays the more important role for certain processes (Ozga *et al.*, 2022).

In Arabidopsis the effect of various Cl-IAA analogues was studied *in vitro*, and 4-Cl-IAA seemed to be among the most active forms in a plant that normally does not possess chlorinated IAAs (Walter *et al.*, 2020). Since the 4-Cl-halogenase has not been identified as yet, Walter *et al.* (2020) transformed Arabidopsis with 5-, 6-, and 7-halogenases deriving from bacteria, and analysed the phenotype of the transgenic plants. As expected, no significant difference was found in the phenotypes of plants grown under control conditions, whereas the addition of Cl-IAA to plants *in vitro* resulted in the inhibition of growth. However, transgenic *Brassica rapa* (Chinese cabbage) plants expressing the same bacterial halogenase genes showed slight alterations in their growth (Neumann *et al.*, 2020). Furthermore, it is not yet clear to what extent 4-Cl-IAA is degraded or conjugated, compared with IAA, both being mechanisms for regulation. Albeit the 4-Cl-IAA methylester and aspartate amide conjugates have been unequivocally identified in pea seeds (Hattori and Marumo, 1972) and quantified later in young developing seeds and pericarp (Magnus *et al.*, 1997), Staswick *et al.* (2005) did not find strong evidence for the enzymatic conjugation of 4-Cl-IAA to amino acids, whereas Walter *et al.* (2020) showed that various chlorinated IAA derivatives were accepted by one GH3 auxin amino acid synthetase *in vitro*.

## Many layers of plant tissues convert the signals to growth responses

Pea contains members of all four auxin receptor clades TIR1, AFB2, AFB4, and AFB6 (Jayasinghege *et al.*, 2019), whereas Arabidopsis lacks the AFB6 clade (Parry *et al.*, 2009), and therefore the characterization of a member of the AFB6 clade is of particular interest for understanding auxin perception. The binding to the receptor of the auxin and a transcriptional inhibitor results in the polyubiquitination of the target, which ultimately leads to its degradation in the 26S proteasome complex of the cell (Box 1). Only then are activators able to initiate auxin-dependent transcription, and this could activate the biosynthesis genes for GAs, and also modify ethylene biosynthesis and response in fruit tissues. Previous work by the same team of authors has shown that during early fruit development, auxin (postulated to be seed derived) stimulates pericarp GA biosynthesis (Ozga *et al.*, 2009), and modifies ethylene biosynthesis and signalling gene expression and response (Johnstone *et al.*, 2005; Jayasinghege *et al.*, 2017). In these cases, the processes are differentially affected by the two auxins. 4-Cl-IAA, and not IAA, stimulates pericarp GA biosynthesis and pericarp growth (Ozga *et al.*, 2009), and the expression of ethylene biosynthesis and signalling genes and ethylene response was differentially modified by the two auxins (Johnstone *et al.*, 2005; Jayasinghege *et al.*, 2017). Also, the GA response is altered by IAA differently from that by 4-Cl-IAA (Johnstone *et al.*, 2005).

The role of auxin receptors in the differential response of these auxins to fruit and seed development was explored in Ozga *et al.* (2022). Rapidly expanding seed tissues had higher auxin receptor gene expression, and this elevated auxin receptor expression facilitated auxin-related developmental processes (Box 2). The physical interaction of pea auxin receptors with Aux-IAAs has been shown using yeast two-hybrid analysis for 4-Cl-IAA in addition to IAA. The transcripts of pea auxin receptor and transcriptional inhibitor genes, as well as genes for auxin biosynthesis, were quantified in individual fruit/seed tissues. This was complemented by hormone analysis (Ozga *et al.*, 2022).

Box 2. **Putative pea pod and seed development**

The tissue-specific auxin and gibberellin levels positively regulate pod development, while ethylene is a negative regulator. Auxins and gibberellins are decreased in deseeded pods, while the addition of auxin can rescue the pericarp growth phenotype, and 4-Cl gives a better response than IAA. For further seed development, the differential transcription of auxin biosynthesis and selected auxin receptors is needed. The figure is based on the contents of fig. 9 of Ozga *et al.* (2022). The pea plant tissue parts are from BioRender (free version: https://biorender.com/; accessed 5 May 2022).

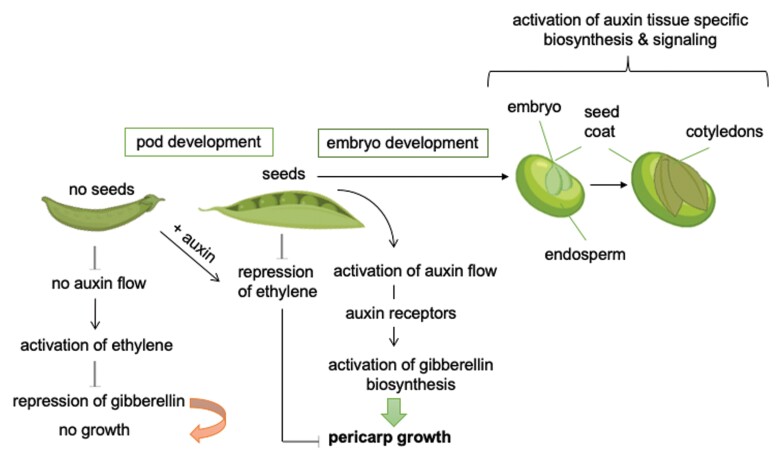

Expression profiles of three different auxin receptor genes in the pericarp were regulated by developing seeds or their absence, probably by different mechanisms (Ozga *et al.*, 2022). Deseeded pericarps were used to determine the effect of the two auxins, IAA and 4-Cl-IAA (as a presumed seed-derived signal), on auxin receptor gene expression, since the authors had shown that all receptors interacted in a auxin-dependent manner in a yeast two-hybrid assay with Aux/IAA proteins. Indeed, the auxins were able to regulate the auxin receptor transcription differently (Box 1). IAA had only short transitory effects on transcription, if any, whereas 4-Cl-IAA had a much stronger response on gene expression. Treatment of pericarps with GA, which also stimulates growth, did not alter the transcriptional response of the auxin receptors assessed. Additionally, the ethylene response was investigated since in the absence of developing seeds, pericarp auxin levels decline (Jayasinghege *et al.*, 2019) and ethylene biosynthesis and evolution increase (Orzáez *et al.*, 1999; Johnstone *et al.*, 2005; Jayasinghege *et al.*, 2017). The reduction in auxin levels leads to reduced GA biosynthesis and increased ethylene action, which facilitates pericarp senescence.

Application of 4-Cl-IAA, but not IAA, completely repressed the ethylene response with respect to pericarp growth and transcriptional regulation of auxin receptors in deseeded pods. Furthermore, 4-Cl-IAA-specific reduction in ethylene responsiveness was associated with the inhibition of ethylene-related induction of pericarp *PsAFB6* expression.

In the developing seeds, the levels of both auxins were high in the seed coat, which can be associated with rapid growth. However, in the later seed filling stage, 4-Cl-IAA was still higher while IAA decreased, indicating a role for 4-Cl-IAA during this developmental growth process. In seedling tissues, auxin receptor transcription was not changed after auxin treatments (Jayasinghege *et al.*, 2019; Ozga *et al.*, 2022), as was the case in the pericarp tisssue, showing tissue-specific responses to these auxins.

## Learning from the combination of methods

The authors attributed the major differences in the responses to the two auxins at least in part to their receptor binding properties, but the transcription of auxin biosynthesis genes has also been altered. Since the enzymes encoded by the *TAR* and *YUC* genes can use both tryptophan and 4-Cl-tryptophan and their respective intermediates as substrates, the differentiation cannot be made on the level of the enzymes.

There is no doubt that this study is an important leap forward towards our understanding of this developmental process. The plethora of methods brought together here could be adapted to pea mutants in the respective pathways and help to further analyse the levels of the two auxins as well as transcripts in a more comprehensive manner in pea pod and seed development. One such candidate could be the *RMS2* pea mutant that is deficient in the AFB4 receptor (Ligerot *et al.*, 2017), or pea mutants in auxin biosynthesis (Tivendale *et al.*, 2012).

Albeit the work does not imply a functional component for application in the field, the latter is obvious from the importance of pea seeds due to their many nutrients in food and fibre technology (reviewed in Kumari and Deka, 2021). If our understanding of pea pod and seed development can be further advanced, it might be possible to use this knowledge to increase yields in the field. Overall, by further understanding how legumes such as pea have adapted their reproductive physiology to the presence of the highly bioactive auxin 4-Cl-IAA, new possibilities in crop management will be revealed and new insights into the basic processes of reproduction may be achieved. The group has already started to transfer the knowledge to other species and showed that 4-Cl-IAA was more effective in a non-legume species, *Triticum aestivum*, to increase grain yield which would allow the use of this auxin as a management tool in another crop (Abeysingha *et al.*, 2021).
